# NIR-driven Smart Theranostic Nanomedicine for On-demand Drug Release and Synergistic Antitumour Therapy

**DOI:** 10.1038/srep14258

**Published:** 2015-09-24

**Authors:** Pengfei Zhao, Mingbin Zheng, Zhenyu Luo, Ping Gong, Guanhui Gao, Zonghai Sheng, Cuifang Zheng, Yifan Ma, Lintao Cai

**Affiliations:** 1 Guangdong Key Laboratory of Nanomedicine, Institute of Biomedicine and Biotechnology, Shenzhen Institutes of Advanced Technology (SIAT), Chinese Academy of Sciences, Shenzhen, 518055, P. R. China; 2 Department of Chemistry, Guangdong Medical University, Dongguan, 523808, P. R. China; 3 University of Chinese Academy of Sciences, Beijing 100049, PR China

## Abstract

Smart nanoparticles (NPs) that respond to external and internal stimulations have been developing to achieve optimal drug release in tumour. However, applying these smart NPs to attain high antitumour performance is hampered by limited drug carriers and inefficient spatiotemporal control. Here we report a noninvasive NIR-driven, temperature-sensitive DI-TSL (DOX/ICG-loaded temperature sensitive liposomes) co-encapsulating doxorubicin (DOX) and indocyanine green (ICG). This theranostic system applies thermo-responsive lipid to controllably release drug, utilizes the fluorescence (FL) of DOX/ICG to real-time trace the distribution of NPs, and employs DOX/ICG to treat cancer by chemo/photothermal therapy. DI-TSL exhibits uniform size distribution, excellent FL/size stability, enhanced response to NIR-laser, and 3 times increased drug release through laser irradiation. After endocytosis by MCF-7 breast adenocarcinoma cells, DI-TSL in cellular endosomes can cause hyperthermia through laser irradiation, then endosomes are disrupted and DI-TSL ‘opens’ to release DOX simultaneously for increased cytotoxicity. Furthermore, DI-TSL shows laser-controlled release of DOX in tumour, enhanced ICG and DOX retention by 7 times and 4 times compared with free drugs. Thermo-sensitive DI-TSL manifests high efficiency to promote cell apoptosis, and completely eradicate tumour without side-effect. DI-TSL may provide a smart strategy to release drugs on demand for combinatorial cancer therapy.

Nanocarrier-based drug delivery has offered great opportunities to promote drug bioactivity and biocompatibility for cancer therapy^1^,^2^. To improve drug delivery and availability, smart vesicles that respond to external stimulations (such as light, magnetic field, ultrasound, and hyperthermia) and internal stimulation (such as reduction/oxidation, pH, and enzyme) have been developing with perfect performances^3^,^4^,^5^,^6^. These stimuli can perfectly implement excellent spatial, temporal, and dosage control (so called spatiotemporal control) for drugs release in tumour tissues with reduced systemic toxicity^7^,^8^. Among these stimuli, temperature exhibits a great potential to release drugs from thermo-sensitive liposomes (TSL), and has been systematically explored in oncology^7^. The underlying mechanism of the controlled drug release from TSL is that the thermo-responsive TSL easily undergo a gel-to-liquid phase transition in the region of their phase transition temperature, the permeability of NPs increases due to the instability of the shell, resulting in drugs spread out rapidly^9^,^10^. Conventional heating techniques, such as water bath (WB), radiofrequency oscillators, and miniature annular-phased array microwave applicators, are major methods to achieve the phase transition temperature at tumour site^11^. But hyperthermia attained by WB is limited by its disadvantages, such as direct contact with body, scald at contact area, and inability to permeate deep tissues^11^,^12^,^13^. Similarly, those strategies of radiofrequency oscillators and microwave applicators are restricted by the high cost and complicated manipulation^14^.

Exploring noninvasive, low-cost, and remote stimuli-triggered drug release for tumour therapy is an optimized approach to acquire improved antitumour efficacy, comparing with traditional temperature stimulation. Currently, near-infrared (NIR) laser induced hyperthermia has been developed to spatiotemporally control the release of drug in NPs^7^,^15^,^16^,^17^,^18^. NIR laser in 650–900 nm regions is an optimal choice providing deep tissue penetration for *in vivo* cancer therapy^19^,^20^. Drugs encapsulated in NPs could be released controllably through NIR laser-induced hyperthermia. It has been reported that NIR photothermal agents, such as fluorescent dyes, gold nanomaterials, carbon nanotubes, and graphene oxide, could strongly absorb NIR laser and induce adequate temperature increase to trigger the rapid drug release in thermo-responsive systems^3^,^21^,^22^,^23^,^24^.

As is well known, cancer therapy is a long-standing, formidable and complicated process. Single strategy such as chemotherapy, photothermal therapy, targeting therapy, and immunotherapy could not eliminate tumours completely^24^,^25^,^26^. Some advanced drug-delivery systems with chemo/photodynamic or immuno/photothermal combination have been reported and manifested great potential for cancer therapy. For instance, theranostic red blood cells modified with iron oxide NPs, Ce6, DOX and PEG have been successfully used for imaging-guided chemo/photodynamic combined therapy^27^. The chitosan-coated hollow CuS NPs assembling immuneadjuvants oligodeoxynucleotides achieved photothermal immunotherapy to cure breast cancer more efficiently than either immunotherapy or photothermal therapy alone^28^.

Briefly, developing NPs with spatiotemporally controlled drug release and synergistic therapy can be an optimal strategy for intratumoural delivery and cancer therapy. It has been reported that Gendicine® (gene therapy medicine), HF10 (oncolytic virus), etc. by intratumoural injection can successfully suppress breast, head and neck, or pancreatic tumour in clinical applications^29^,^30^. However, cancer combination therapy using more than one drug is usually hampered by quick tumour clearance, non-uniform distribution and instability of drugs^31^. In order to achieve maximal synergistic effects, co-encapsulating two or more drugs in TSLs is developed to obtain highly-stable co-delivery of drugs with optimal dose. Here, doxorubicin (DOX)/indocyanine green (ICG) co-loaded thermo-sensitive liposomes (DI-TSL) was synthesized through the film hydration via strong sonication followed by extrusion technique^9^,^10^. This new strategy integrated real-time tracking, NIR laser-driven drug release, chemo/photothermal therapy, and afforded optimized efficacy to inhibit tumour growth through enhanced passive targeting and combinatorial therapeutic mechanisms in tumour intracellular circumstance and extracellular matrix (Fig. 1). Notably, DOX was an agent for chemotherapy, ICG was a photothermal agent to induce hyperthermia, and 1, 2-distearoyl-sn-glycerophosphocholine (DPPC) and 1, 2-dimyristoyl-sn-glycero-3-phosphocholine (DMPC) were thermo-responsive lipids (phase transition temperature approximately 43 °C) for hyperthermia driven NPs destruction^32^. Benefited from the formulation, the DI-TSL could be served as an ideal fluorescent imaging probe for dynamically monitoring the metabolism and consumption of drugs/photosensitizer, and regulating drug release by laser triggering for chemo-photothermal combination therapy. The physiochemical characters were systematically investigated to explore the mechanism for drug release of DI-TSL. Fluorescence (FL) of DOX and ICG in DI-TSL was monitored to demonstrate subcellular localization, NIR laser-driven drug release, and metabolic distribution. The cytotoxic effects of DI-TSL, which combined controllable drug release, chemotherapy and photothermal therapy, were gradually evaluated in MCF-7 cells. Moreover, the antitumour efficacy of DI-TSL *in vivo* through intratumoural injection was further appraised in comparison with single strategy.

## Results

### Synthesis and characterization of the temperature-sensitive DI-TSL

To integrate the thermo-responsive properties of lipids, FL properties of DOX/ICG, and chemo/photothermal therapy of DOX/ICG, temperature-responsive DI-TSL was assembled using DPPC, DMPC, DSPE-PEG, cholesterol, DOX and ICG^33^,^34^. TSL (composed of DPPC, DMPC, DSPE-PEG and cholesterol) was utilized to co-encapsulate clinical anticancer drug DOX and theranostic agent ICG. TSL can undergo an irreversible phase transition to achieve rapid release of encapsulated drugs as the temperature rising higher than 43 °C^35^. The morphology obtained by transmission electron microscopy (TEM) demonstrated that DI-TSL exhibited excellent monodispersity, generally spherical shape and uniform size distribution (Fig. 2a). Particle size and zeta potential were detected by dynamic light scattering (DLS) and shown in Supplementary Table 1. The hydrodynamic size and surface potential of DI-TSL was 43.8 nm and −23.5 mV, respectively, and more than 90% DI-TSL in number distribution were 20–50 nm. Previous works had proved that NPs with small size were much easier to be internalized by tumour cells and could penetrate into deeper areas of tumours for cancer therapy^36^. Compared with commercial heat-sensitive liposome (ThermoDox®, ~140 nm)^37^, DI-TSL with smaller size (43.8 nm) showed great potential to penetrate into deep tumours for efficient tumour treatment. ICG loaded TSL (ICG-TSL) was also synthesized with similar size (42.2 nm) and surface potential (−26.5 mV). Drug encapsulating efficiency (EE) and loading efficiency (LE) were crucial factor for their clinical application^38^. As shown in Supplementary Table 1, the EE or LE of DOX was 27.1% and 3.5% for DI-TSL, the EE or LE of ICG was 28.1% and 3.9% for DI-TSL, and the EE or LE of ICG in ICG-TSL was 40.9% and 6.4%.

A size stability assay of DI-TSL was evaluated in ultrapure water, phosphate buffer saline (PBS), 10% (v/v) plasma/heparin in PBS, and fetal bovine serum (FBS) for 5 d at 37°C, respectively (Fig. 2c)^39^. The DI-TSL dissolving in ultrapure water and PBS remained the similar size within 5 d (about 43 nm), indicating a long-term stability in these solutions. And the stability for protein binding was investigated by dissolving the DI-TSL in plasma/heparin solution or FBS. The size of DI-TSL increased 20–30 nm and remained stable for 5 d, implying that the protein binding of plasma has no impact on their stability. The results demonstrated that DI-TSL maintained excellent size stability without precipitation or phase separation in ultrapure water, PBS, plasma/heparin in PBS, and FBS. Notably, ICG has been widely used as an NIR FL probe for diagnosis and therapy due to substantial absorption, FL in the NIR wavelength region, and effective photothermal response, but its further application was hindered owing to unstable optical property, quick degradation and metabolism limitation^40^,^41^. Therefore, the ICG FL stability of free ICG and DI-TSL was investigated by comparison. After 2 d, The ICG FL intensity of DI-TSL in ultrapure water remained at 89% and even above 82% after 5 d, while the ICG FL intensity of free ICG degraded to about 73% of initial intensity and 51% after 5 d (Fig. 2d). Furthermore, DI-TSL was dissolved in PBS and FBS for 5 d to illustrate the superior ICG FL stability. The results indicated that the encapsulation of ICG into DI-TSL significantly enhanced its FL stability. The FL stability of ICG in different solutions and temperature conditions was investigated (Supplementary Fig. 1). ICG after TSL encapsulation in ultrapure water, PBS and FBS solutions exhibited consistent FL spectral characters of free ICG in ultrapure water (Supplementary Fig. 1a). The ICG FL of DI-TSL was well maintained at 4, 25, 37, 43, 70 °C in ultrapure water, PBS or FBS, indicating that ICG FL of DI-TSL in ultrapure water, PBS or FBS was not influenced by temperature (4–70 °C) (Supplementary Fig. 1b).

ICG is a member of cyanine that generally consists of two heterocyclic units connected with linear alkene units^42^. When irradiated by NIR laser, ICG molecules absorb luminous energy and reach to the high vibration energy level of excited state^43^. Then ICG molecules generate thermal energy and act out by hyperthermia and photothermal effects during the deactivation process. Therefore, the temperature increasing profiles under laser irradiation (808 nm, 1 W/cm^2^) *in vitro* was monitored to evaluate the photothermal efficiency of DI-TSL. The temperature of free ICG, ICG-TSL and DI-TSL aqueous solution showed a quick temperature increase within 5 min, and the maximal temperature at 8 min increased to 66.9 °C, 69.6 °C and 69.2 °C, respectively. However, the maximal temperature of PBS and free DOX solution only increased 4.9 °C and 5.1 °C (Fig. 2e). The photothermal efficiency of ICG encapsulated in NPs was enhanced slightly, which was consistent with previous reports that ICG-containing NPs was more efficient to trigger laser-dependent temperature increase than free ICG^36^. And NIR laser-induced high-temperature (up to 69.2 °C) was far above 43 °C, which was high enough to transform the thermo-sensitive lipids of the DI-TSL from solid to liquid. This indicated that NIR laser was an effective stimulus to ‘open’ DI-TSL. When laser shut off after 5 min irradiation, the temperature of free ICG, ICG-TSL and DI-TSL aqueous solution rapidly decreased from 66.8 °C, 67.6 °C and 68.6 °C to 40.2 °C, 38.2 °C, and 39.9 °C within 90 s, respectively (Supplementary Fig. 2).

We further tested the DOX release of DI-TSL under different temperature stimulations. DI-TSL in 43 °C WB showed a faster drug release than in 37 °C WB, which released 84% DOX after 6 h and 98% after 48 h. While DI-TSL kept in 37 °C WB only released 27% after 6 h and 43% after 48 h (Fig. 2f, Supplementary Fig. 3a). Compared with DOX release, ICG release showed the similar trends, demonstrating excellent co-encapsulation of DOX and ICG (Supplementary Fig. 3b). It was worth mentioning that the phase transition temperature of DI-TSL was 43 °C, which was away from normothermia (37 °C) in comparison with the phase transition temperature of commercial ThermoDox (39 °C), resulting in 27% DOX release of DI-TSL and 100% DOX release of ThermoDox at 37 °C after 6 h (Fig. 2f, Supplementary Fig. 3a)^37^,^44^. We further investigated the release of DOX triggered by remote NIR laser (808 nm, 1 W/cm^2^, 5 min). DI-TSL treated with laser exhibited a greatly enhanced drug release. DOX released from DI-TSL was boosted at 1 h from 7% to 36% following 5 min laser irradiation. NIR laser triggered DOX release was observed when the NIR laser was repeated at 3 h and 5 h, in which DOX release was increased from 47% to 71% and from 75% to 93%, respectively. Finally, 94% DOX was released after 6 h (Fig. 2g), which was about 3 times higher than DI-TSL without laser treatment, indicating that NIR laser would also trigger drug release from DI-TSL effectively. FL of DOX and ICG in the DI-TSL was studied by the *in/ex vivo* imaging system to further study the drug release affected by laser irradiation (Fig. 2h). The results demonstrated that after laser stimulation (808 nm, 1 W/cm^2^, 5 min), the FL of DOX was very significantly increased. It was probably because that high DOX concentration within DI-TSL brought about self-quenching of DOX FL^45^. And when DI-TSL was ‘opened’ after laser stimulation, DOX released from the ‘opened’ DI-TSL led to a significant increase of DOX FL. The break of DI-TSL was confirmed by TEM and DLS measurement (Fig. 2b) after laser irradiation, which demonstrated that DI-TSL was controllably-opened under NIR laser irradiation by the way of smash, burst or swell. Owing to the photo-bleaching properties of ICG, the irradiation decreased the ICG FL of DI-TSL and free ICG to about 4% and 7% of initial intensity^37^,^38^. The mechanism of NIR laser-triggered drug release was that ICG molecules transferred luminous energy of NIR laser to thermal energy^43^, and the local temperature within DI-TSL rapidly increased to over 43 °C. Then phase transition took place on TSLs, and encapsulated drugs were rapidly released to activate enhanced chemotherapy.

### NIR laser-driven endosome disruption and intracellular DOX release

The intracellular distribution of NPs and NIR laser-induced endosomal disruption in cells was tested utilizing confocal laser scanning microscope (CLSM). To avoid overlapped FL signal between DOX and the Lysotracker, we conducted ICG-TSL for investigation instead of DI-TSL. Without laser irradiation, the FL of Lysotracker and ICG were co-localized, implying that ICG-TSL was mainly localized in endo/lysosomal compartments. After NIR laser irradiation (808 nm, 1 W/cm^2^, 5 min), ICG FL could be clearly detected as more diffused spots in the cytosol compared with the green FL emanated by marked endo/lysosomes (Fig. 3a). This indicated the successful endosomal escape of ICG-TSL and ICG diffusion into the cytosol, which was consistent with previous reports that photothermallly induced heat by reduced graphene oxide or magnetic iron oxide NPs could trigger the endosomal escape of drugs^3^,^46^. Notably, the green FL emanated by endo/lysosomes initially existed in the cytosol was in punctuate distribution but was conglomerated after laser irradiation, which was probably caused by hyperthermia-induced endo/lysosomal disruption. Simultaneously, the amount of DOX released from DI-TSL, reflected by the FL of the whole cell, was evaluated via flow cytometry. The results demonstrated that the cellular uptake obtained in DOX group and DOX + laser group was similar, implying that NIR laser did not affect the cellular uptake of DOX (Fig. 3b,c). However, laser irradiation (808 nm, 1 W/cm^2^, 5 min) significantly increased the DOX FL of the whole cell in DI-TSL + laser group, indicating the release of DOX in DI-TSL to the cytosol could be significantly improved through laser intervention (Fig. 3b,c).

### Increased cytotoxicity by NIR laser-driven DOX release

To directly investigate the photothermal efficiency of NPs, the viability of MCF-7 cells after being exposed to ICG-TSL with various ICG concentrations plus same dosage laser irradiation was assessed (Fig. 4a). The results showed that 88% of cells were still viable even the ICG concentration was increased to 48 μg/mL. The cytotoxicity of DOX, DOX + laser, DI-TSL, and DI-TSL + laser to MCF-7 cells was further investigated respectively (Fig. 4b). The DI-TSL or free DOX were immediately washed away after incubation in the experiments. In the case of free DOX, there was no obvious cytoxicity difference with or without NIR laser irradiation, indicating that the efficacy of DOX was not affected by NIR laser (Fig. 4b). DI-TSL showed a little lower cytotoxocity than free DOX. Apparently, DI-TSL + laser caused a better effect to kill MCF-7 cells than DI-TSL under high DOX concentration (6 μg/mL), which was probably because the relative high ICG concentration induced hyperthermia driven endosomal disruption and the opening of DI-TSL in the mean time, and DOX quickly released to kill MCF-7 cells by synergistic effects of abrupt drug release and chemo/photothermal therapy. However, under lower DOX concentrations (2 μg/mL and 4 μg/mL), the ICG with lower concentration cannot induce sufficient hyperthermia to ‘open’ the DI-TSL for rapid DOX release or directly kill MCF-7 cells by heat (Fig. 4b). The enhanced cytotoxic effects driven by NIR laser was further evaluated through cell apoptosis analysis (Fig. 4c). MCF-7 cells were treated with DOX, DOX + laser, DI-TSL, or DI-TSL + laser for 4 h and then labeled with PI and Annexin V. Double stained cells were regarded as late apoptotic/necrotic cells. The results showed that cell late apoptosis/necrosis was more efficiently induced by DI-TSL + laser (40%) compared with cells treated with DOX (27%), DOX + laser (29%), and DI-TSL (20%). The results evidently illustrated that NIR laser irradiation could obviously promote the effect of DI-TSL on apoptosis and cell death.

### *In vivo* biodistribution of DI-TSL

Concerning whether DI-TSL distributes in tumours/organs or clear out from the body after intratumoural injection, the biodistribution of DI-TSL was investigated by *in/ex vivo* imaging system utilizing the FL of DOX and ICG. The FL images and quantitative analysis of MCF-7 tumour-bearing mice were obtained at 0 h, 16 h, 24 h, and 48 h after intratumoural injection of free DOX + ICG, or DI-TSL. The ICG FL intensity in tumour site of mice injected with free ICG quickly decreased to 49% of initial FL intensity at 16 h, and 9% of initial FL intensity at 48 h (Fig. 5a,b). However, the ICG FL intensity of tumour injected with DI-TSL still retained 77% at 16 h and 43% at 48 h, implying that DI-TSL could be dramatically retained at tumour site. Free DOX was quickly cleared from the tumour and the DOX FL intensity decreased to 9% of initial intensity at 48 h (Fig. 5a,b). It was worth mentioning that the high DOX concentration within DI-TSL brought about self-quenching phenomena, so no obvious DOX signal could be immediately observed after injection of DI-TSL (Fig. 5c,d). The slow release of DOX from DI-TSL over time led to the reappearance and slow increase of DOX FL in tumour sites (Fig. 5c,d). The mice treated with DI-TSL 48 h after injection was sacrificed and major organs were collected for *ex vivo* imaging analysis (Supplementary Fig. 4). The data shown in DOX + ICG group exhibited that free ICG mainly accumulated in liver and kidneys, and could hardly be detected in the tumour. Free DOX was almost cleared away from mice body 48 h after intratumoural injection, and only few DOX was discovered in liver and tumour. On the other hand, DI-TSL obviously increased the reservation of ICG and DOX in tumour. ICG and DOX FL signals could be apparently detected in tumour tissues, followed by kidneys, and liver. And the averaged FL intensity of ICG or DOX at tumour site in DI-TSL group was about 7 times or 4 times higher than that in DOX + ICG group (Supplementary Fig. 4b,d). It was obvious from the results that DI-TSL could significantly improve the retention of ICG and DOX in the tumour through encapsulation of nanocarriers. It deserved noting that in DI-TSL group, DOX FL signals in the major organs of mice were relatively weak because of relatively low dose of DOX accumulated in these organs except for the tumour.

### NIR laser-driven DOX release *in vivo*

The NIR laser-mediated temperature increase *in vivo* was measured 16 h after intratumoural injection of PBS, free DOX, ICG-TSL and DI-TSL, respectively. And the final temperature in each group after 5 min laser irradiation was recorded using infrared imaging camera (Fig. 6a). After laser irradiation (808 nm, 0.5 W/cm^2^, 5 min), the temperature of tumours injected with ICG-TSL or DI-TSL rapidly rose to 56.2 °C and 56.8 °C, which could trigger efficient drug release (above 43 °C) and exceed irreversible tumour tissue damage^47^. However, the maximal temperature of tumours injected with PBS or free DOX for the same laser irradiation was below 43 °C and ineffective to irreversibly damage tumour tissues^48^,^49^. Additionally, the opening of DI-TSL and drug release *in vivo* was explored by the triggering of NIR laser-induced hyperthermia (Fig. 6b,c). The ICG FL intensity of DI-TSL around the tumour reduced to 75% of initial FL intensity 16 h after post-injection, and quickly decreased to 31% after laser irradiation (808 nm, 0.5 W/cm^2^, 5 min) caused by the photo-bleaching of ICG (Fig. 6b,c)^50^. The ICG FL intensity in tumour at 48 h reduced to 5% of initial FL intensity treated with DI-TSL + laser, which was much lower compared with 43% that treated with DI-TSL without laser irradiation (Figs 5b and 6c). The results indicated that remote NIR laser-driven hyperthermia could ‘open’ DI-TSL and induce a faster metabolism of ICG in tumour tissues. Moreover, the DOX FL intensity in the tumour was very weak at 0 h and 16 h because of initial self-quenching (Fig. 6b,c). After laser irradiation at 16 h, DOX could immediately release from DI-TSL and the DOX FL intensity in the tumour increased 7 times compared with the FL intensity before laser irradiation. The results further proved that NIR laser could ‘open’ DI-TSL by spatiotemporal control and stimulate drug release on demand.

### *In vivo* apoptosis imaging and enhanced antitumour therapy

**Figure 1:**
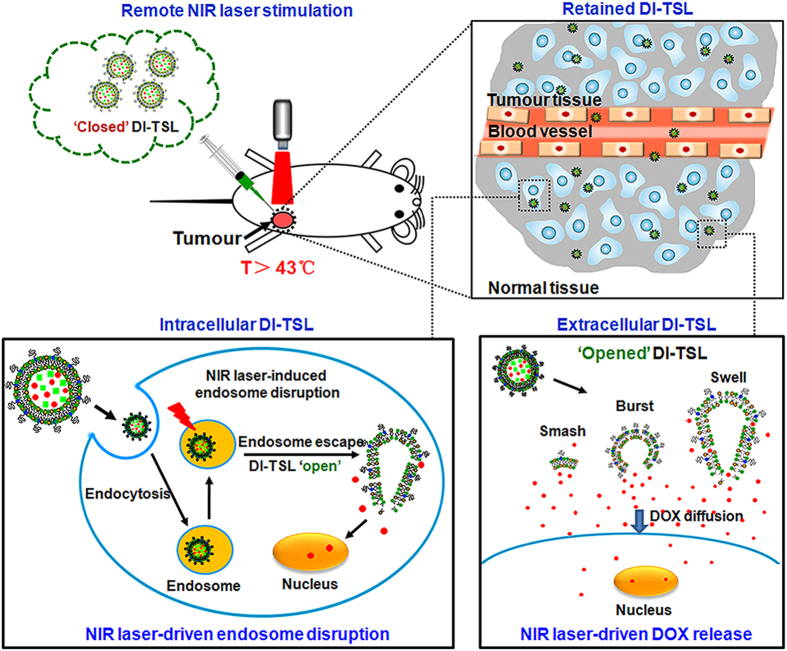
A scheme showing the mechanism of remote NIR-triggered DOX release and further cytotoxicity in tumour. The DI-TSL are treated with remote NIR laser (808 nm, 0.5 W/cm^2^, 5 min) after the injection, and kill cancer cells in different ways: 1) Intracellular DI-TSL escape from cell endosomes by NIR laser induced endosomal disruption, and DOX release from ‘opened’ DI-TSL and enter the cytosol after cellular uptake. 2) Extracellular DI-TSL immediate released DOX through smash, burst, and swell, and DOX diffuses into the tumour along a high concentration gradient, attacking tumour cells.

**Figure 2:**
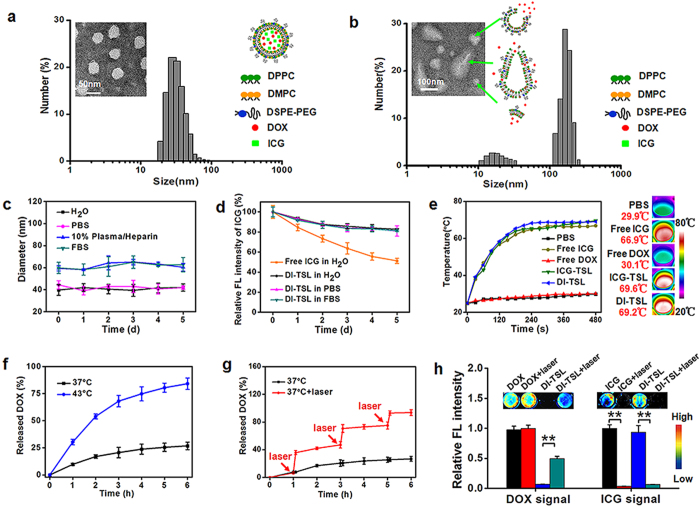
Characterization of DI-TSL and NIR laser-driven DOX release. (**a**) Size distribution, TEM images and schematic representation of DI-TSL (Scale bar = 50 nm). (**b**) Size distribution and TEM images of DI-TSL after laser irradiation (808 nm, 1 W/cm^2^, 5 min), and schemes showing smash, burst, and swell of DI-TSL (Scale bar = 100 nm). (**c**) Size stability of DI-TSL in ultrapure water, PBS, 10% plasma/Heparin in PBS, and FBS within 5 d. (**d**) ICG FL stability of free ICG in ultrapure water, and ICG FL stability of DI-TSL in ultrapure water, PBS, and FBS within 5 d. (**e**) Temperature rising profiles of PBS, free ICG, free DOX, ICG-TSL and DI-TSL under continuous laser irradiation. (**f**) DOX release curves of DI-TSL at 37 °C or 43 °C water bath (WB). (**g**) DOX release profiles of DI-TSL in 37 °C without or with laser irradiation (808 nm, 1 W/cm^2^, 5 min). The red arrows indicated the time point of laser irradiation. (**h**) Evaluation of DOX signals in DOX, DOX + laser, DI-TSL and DI-TSL + laser groups, and ICG signals in ICG, ICG + laser, DI-TSL and DI-TSL + laser groups (n = 3). **P < 0.01.

**Figure 3:**
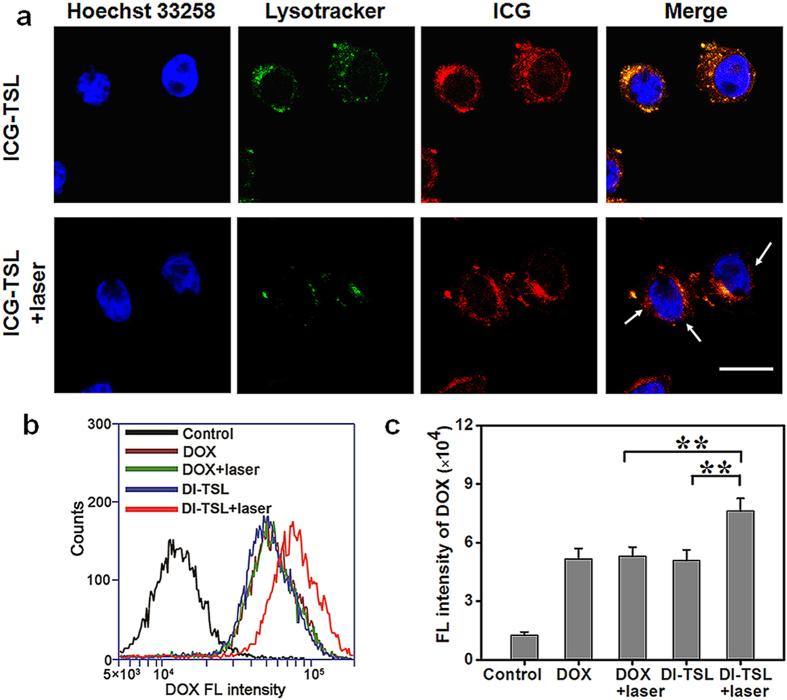
NIR laser-driven drug release of DI-TSL in MCF-7 cells. (**a**) CLSM images of MCF-7 cells treated with ICG-TSL without (top) or with (bottom) NIR laser irradiation (808 nm, 1 W/cm^2^, 5 min). Nuclei were stained with Hoechst 33258 (blue), endo/lysosomes were stained with Lysotracker (green), and red was the FL of ICG in the ICG-TSL. (**b**) Flow cytometry histogram profile of DOX FL in MCF-7 cells treated with DOX, DOX + laser, DI-TSL, or DI-TSL + laser. (**c**) Mean FL intensity analysis for flow cytometry of MCF-7 cells after incubation with DOX, DOX + laser, DI-TSL, or DI-TSL + laser (n = 3). **P < 0.01. Scale bar = 20 μm.

**Figure 4:**
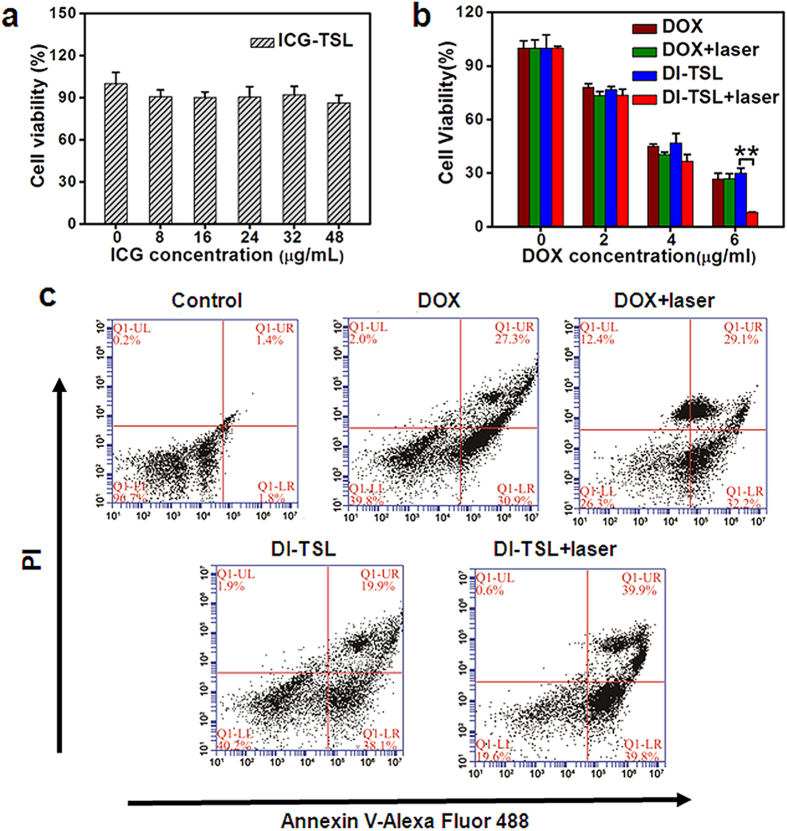
NIR laser-driven drug release for increased cellular cytotoxicity. (**a**) Cell viability of MCF-7 cells treated with ICG-TSL of various ICG concentrations and the same NIR laser irradiation (808 nm, 1 W/cm^2^, 5 min) (n = 3). (**b**) Cell viability of MCF-7 cells treated with free DOX or DI-TSL containing various DOX concentrations without and with NIR laser irradiation (808 nm, 1 W/cm^2^, 5 min). The DI-TSL or free DOX were immediately washed away after incubation in the experiments. (n = 3). **P < 0.01. (**c**) Flow cytometry analysis of MCF-7 cells after incubation with free DOX, DOX + laser, DI-TSL, or DI-TSL + laser. Double stained cells were considered as late apoptotic/necrotic cells.

**Figure 5:**
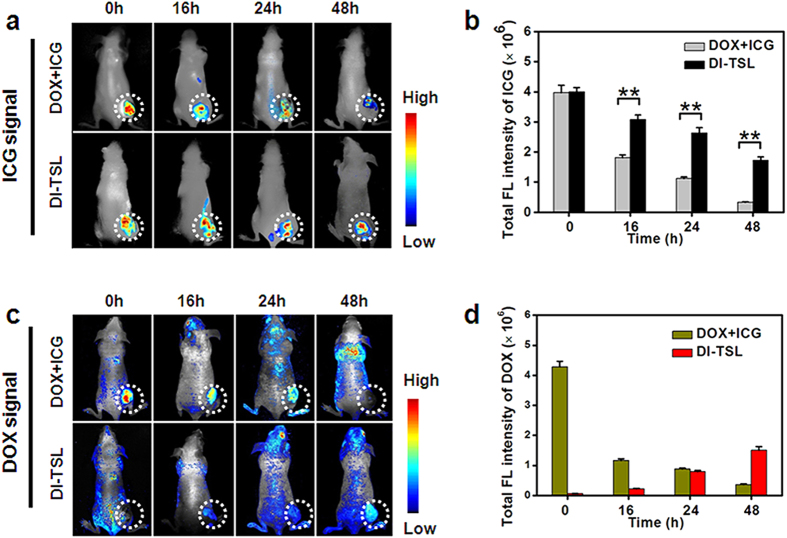
*In vivo* FL imaging of DOX or ICG in nude mice bearing MCF-7 tumours after intratumoural injection of DOX + ICG, or DI-TSL. (**a**) Time-lapse ICG FL in nude mice within 48 h after injection of DOX + ICG or DI-TSL (tumours were marked with white-dotted circles). (**b**) Quantified total FL intensities of ICG at different time points (n = 3). **P < 0.01. (**c**) Time-lapse DOX FL *in vivo* within 48 h after injection of DOX + ICG or DI-TSL (tumours were marked with white-dotted circles). (**d**) Quantified total FL intensities of DOX at different time points (n = 3).

**Figure 6:**
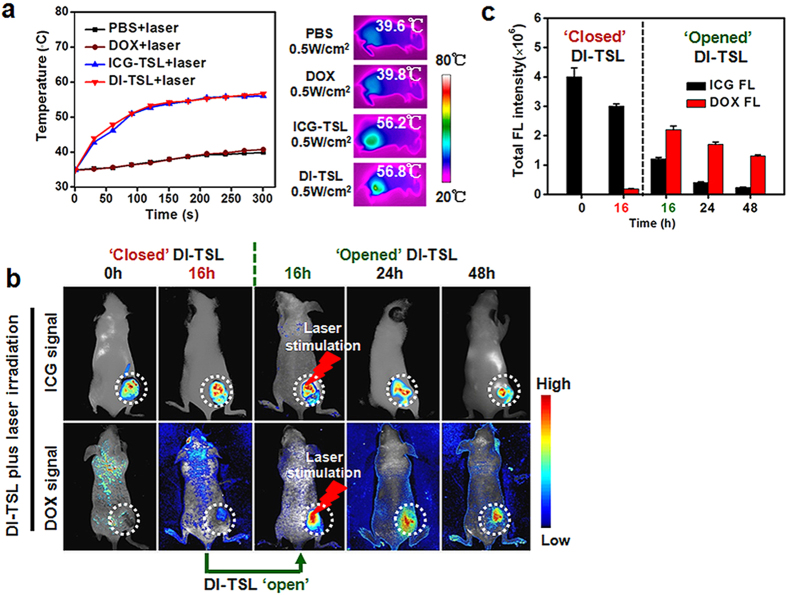
NIR laser-induced temperature increase in MCF-7 tumour tissues, and *in vivo* imaging of nude mice bearing MCF-7 tumours showing laser-driven DOX release. (**a**) Temperature increasing profiles of laser-irradiated (808 nm, 0.5 W/cm^2^, 5 min) tumour tissues 16 h after injection of PBS, DOX, ICG-TSL, or DI-TSL, and indicated infrared thermo-graphic maps of mice after 5 min irradiation. (**b**) Time-lapse FL images of DOX and ICG after intratumoural injection of DI-TSL + laser. The laser was treated 16 h after the injection (tumours were marked with white-dotted circles). (**c**) Semi-quantitative FL intensities of DOX and ICG around the tumour at the time points as indicated.

**Figure 7:**
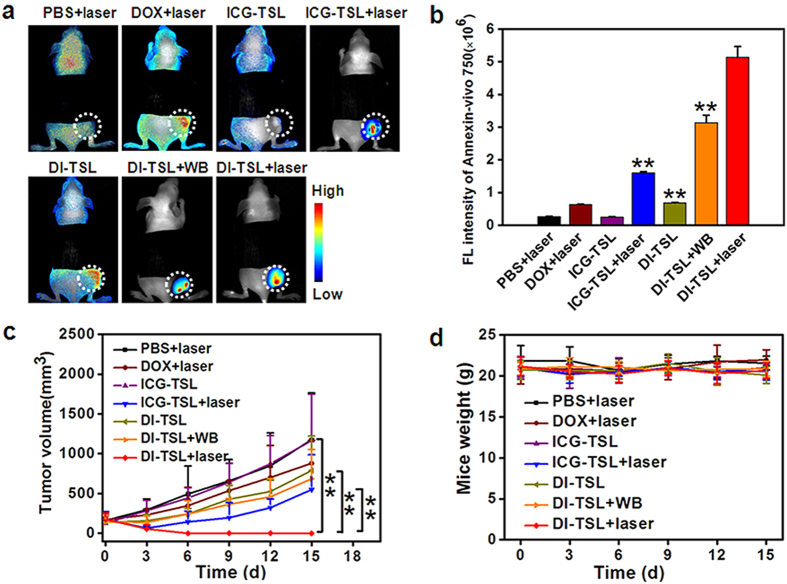
*In vivo* apoptosis imaging showing the accumulation and retention of apoptosis marker Annexin-Vivo 750 in tumours, and synergistic strategies of DI-TSL for tumour therapy. (**a**) *In vivo* apoptosis images 24 h after Annexin-Vivo 750 injection for different treatment as indicated (tumours were marked with white-dotted circle), showing apoptotic tumour cells in the tumour induced by different treatments. (**b**) Quantitative FL intensities of Annexin-Vivo 750 around the tumour (n = 3). **P < 0.01, compared with DI-TSL + laser. (**c**) MCF-7 tumour growth profiles of nude mice in different groups after treatment (n = 5). **P < 0.01. (**d**) Weight profiles of MCF-7 bearing mice after treatment as indicated.

Annexin-Vivo 750 could bind to apoptotic cells and reveal information about immediate cell death induced by drug release^35^,^51^. Annexin-Vivo 750 was intravenously injected 48 h after NIR irradiation to demonstrate the immediate effect of drug release from NPs on cell viability *in vivo*. The FL images of Annexin-Vivo 750 were collected (excited at 740 nm; emission collected at 780-850 nm) by *in/ex vivo* imaging system 24 h after the post-injection. Because of relate weak signals of ICG, the ICG fluorescence was regarded as background FL and was eliminated when analyzing the FL of Annexin-Vivo 750. The DI-TSL + laser (808 nm, 0.5 W/cm^2^, 5 min) group showed the maximum accumulation of Annexin-Vivo 750 at tumour site, indicating the best efficacy to induce cell apoptosis, followed by DI-TSL + WB, ICG-TSL + laser, DI-TSL, and DOX + laser (Fig. 7a,b). Moreover, the very significant difference between DI-TSL + laser and DI-TSL + WB/ICG-TSL + laser groups demonstrated that combinatorial strategies were more superior to single controllable drug release, chemotherapy or photothermal therapy. DI-TSL + laser performed an increased efficacy to promote apoptosis of tumour cells due to the integration of controllable drug release, chemotherapy and photothermal therapy in tumour intracellular circumstance and extracellular matrix (Fig. 1). The antitumour effect of DI-TSL *in vivo* was further estimated in nude mice bearing MCF-7 tumours. As shown in Fig. 7c, tumours treated with PBS + laser or ICG-TSL grew rapidly, suggesting that the growth of MCF-7 tumours was not affected by laser irradiation or ICG-TSL. Tumours treated with ICG-TSL + laser recurred within 6 d, and the tumour growth was slightly suppressed by DOX + laser, DI-TSL and DI-TSL + WB with an averaged tumour size of 880 mm^3^, 789 mm^3^ and 685 mm^3^ on day 15, respectively (Fig. 5c, Supplementary Fig. 5). Traditional chemotherapy usually needs high dosage of DOX (5 mg/kg) and multiple injections to inhibit tumour growth^52^,^53^. The DOX dosage (0.5 mg/kg) used in these treatments was insufficient to cure tumour^36^. Surprisingly, tumours treated with a single injection of DI-TSL plus laser irradiation (808 nm, 0.5 W/cm^2^, 5 min) led to a complete tumour inhibition without recurrence on 15 d, which demonstrated that the low dosage of DOX and laser treatment was enough to eradicate tumours integrating efficient drug release, chemotherapy and photothermal therapy, which was consistent with the Annexin-Vivo 750 FL imaging results *in vivo*. Furthermore, the loss of body weight was analyzed to indicate treatment-induced toxicity (Fig. 7d). Nude mice bearing MCF-7 tumours in all the indicated groups showed a slight increased body weight, which was not significantly different from the control group (PBS + laser). The DOX dosage used in the indicated treatment was well tolerated. H&E staining images of major organs further indicated that all the treatments containing DOX were biocompatible and safe to nude mice (Supplementary Fig. 6). DI-TSL + laser treatment, which integrated real-time tracing, controllable drug release, and chemo/photothermal therapy, achieved an optimal strategy for antitumour therapy without adverse effect or resistance.

## Discussion

A variety of technologies have recently emerged for NIR laser-induced drug release^7^,^15^,^16^,^17^. However, most of these strategies were hampered by invisible distribution and insufficient efficacy for antitumour treatment. The theranostic DI-TSL integrated a series of components to assemble modified NPs, and offered a smart combinatorial mechanism for effective antitumour therapy. In addition, ICG was applied first time as a trifunctional agent for optical imaging, photothermal therapy, and photothermal agent for hyperthermia-triggered drug release. Remarkably, FL was abundantly used to directly observe the drug release, biodistribution and antitumour efficacy of DI-TSL, which established a completely visible system to evaluate the antitumour efficacy of nanomedicines.

In summary, DOX/ICG-loaded temperature-responsive DI-TSL were synthesized with excellent size distribution and stability. DI-TSL perfectly integrated the thermo-responsive properties of thermo-sensitive lipids, FL imaging properties of DOX/ICG and chemo/photothermal properties of DOX/ICG in a single nanocomposite. DI-TSL exhibited excellent temperature response and NIR laser-controlled drug release triggered by sufficient heat (above 43 °C) both *in vitro* and *in vivo*, which successfully transformed the stimulus from heat to NIR laser. With external NIR laser stimulation, DI-TSL internalized in MCF-7 cell induced local hyperthermia, endosomal disruption, rapid drug release, and caused enhanced effect to kill cancer cells. Moreover, taking advantage of the DOX/ICG FL, the biodistribution of DI-TSL and DOX release driven by NIR laser *in vivo* was directly observed through *in/ex vivo* imaging system. Due to high dosage of DI-TSL reserved in tumour tissues, NIR laser-driven drug release, and high photothermal efficacy, MCF-7 tumours treated with DI-TSL + laser were completely eradicated. Hence, we expected that DI-TSL would represent a smart and versatile strategy to promote antitumour efficacy through synergy of TSLs, enhanced chemotherapy, and photothermal therapy.

## Methods

### Materials

(1, 2-distearoyl-sn-glycero-3-phosphoethanolamine-N-maleimide (polyethylene glycol 2000) (DSPE-PEG), 1, 2-dipalmitoyl-sn-glycero-3-phosphocholine (DPPC), 1, 2-dimyristoyl- sn-glycero-3-phosphocholine (DMPC) were purchased from Avanti (USA). Cholesterol, indocyanine green (ICG), 3-(4, 5-dimethylthiazol-2-yl)-2, 5-diphenyltetrazolium bromide (MTT), hematoxylin and eosin (H&E) were acquired from Sigma-Aldrich (USA). Doxorubicin (DOX) was obtained from Jinhe (China). Alexa Fluor488 Annexin-V and Propidium Iodide (PI), and Lysotracker Green DND-26 were obtained from Life Technologies (USA). Amicon ultra-4 centrifugal filter with a molecular weight cutoff of 10 kDa was purchased from Millipore (Germany). RMPI 1640 medium, fetal bovine serum, trypsin EDTA, penicillin and streptomycin were obtained from Hyclone (USA).

### Preparation of DI-TSL

DI-TSL was fabricated using the film hydration via strong sonication followed by an extrusion technique. Briefly, DPPC, DMPC, DSPE-PEG, ICG, DOX and cholesterol with a mass ratio of 50: 10: 10: 40: 10: 30 were placed in a round-bottom flask and dissolved in an ethanol/chloroform mixture. The organic solvent was removed with a rotavapor to generate a thin film on the glass vial. Then the film was hydrated with ultrapure water by 5 min strong sonication (VCX130, Ultrasonics, USA). Next, the DI-TSL was acquired by extrusion through 200 nm and 100 nm polycarbonate filters (five times each). Finally, the DI-TSL was washed three times using Ultra-4 centrifugal filter. The same procedures were utilized to synthesize ICG-loaded ICG-TSL.

### Characterization of DI-TSL

The size, surface potential and size distribution of DI-TSL and ICG-TSL were measured by DLS detector Malvern Zetasizer (Malvern, USA) and TEM (F20, Techni, USA). The TEM samples were prepared by placing a drip of DI-TSL onto 300-mesh copper-grid (Zhongjingkeyi, China), and a drip of 2 wt% phosphotungstic acid was dropped on the sample for negative staining, then keep the sample at room temperature overnight for drying. The encapsulating efficiency (EE) and loading efficiency (LE) of DOX and ICG encapsulated in DI-TSL and ICG-TSL were detected as follows: isolating the fresh NPs from aqueous suspension medium by ultra-centrifuge (25,000 r/min, 30 min) (MAX-XP, Beckman, USA). Suspension liquid containing non-loaded drugs was removed. Then the concentration of ICG and DOX encapsulated was determined by FL spectrometry (F900, Edinburgh Industries, UK). The EE and LE were detected through the following formula: EE (%) = (weight of loaded drug)/(weight of initially added drug) × 100; LE (%) = (weight of loaded drug)/(total weight of NPs) × 100. The FL spectra of free ICG, free ICG aqueous solution, DI-TSL aqueous solution, and DI-TSL in FBS were acquired by FL spectrometry with excitation wavelength at 740 nm. The size stability of the DI-TSL within ultrapure water, PBS, 10% (v/v) plasma/heparin in PBS, and fetal bovine serum (FBS) for 5 d at 37 °C. The size was obtained by Malvern zetasizer. The FL stability of free ICG was performed in ultrapure water, and the FL stability of DI-TSL was performed in ultrapure water, PBS and FBS. ICG FL was acquired by FL spectrometry.

### Drug release of DI-TSL *in vitro*

Drug release profiles were acquired according to the following steps. Briefly, 1000 μL DI-TSL aqueous solution was added into centrifugal tubes, and was kept at 37 °C water bath (WB), 43 °C WB, or 37 °C plus laser irradiation (808 nm, 1 W/cm^2^, 5 min) every 2 h, respectively. 10 μL of each samples were collected at corresponding time and added with 10% v/v triton-100 to disrupt the NPs. The profiles were obtained by fluorescence spectroscopy. Released DOX were calculated according to the below formula: Released DOX (%) = (F_t_–F_0_)/(F_100_–F_0_) × 100. Herein, F_0_ represent for the initial DOX FL of DI-TSL aqueous solution at the start point, F_t_ represent for the DOX FL at indicated time point, and F_100_ represent for the DOX FL of DI-TSL after Triton-100 treatment. ICG release profiles at 37 °C and 43 °C were obtained by the same method.

### NIR laser-induced temperature increase *in vitro*

1 mL of PBS, free ICG (ICG: 19.3 μg/mL), free DOX (DOX: 5 μg/mL), ICG-TSL (ICG: 19.3 μg/mL) and DI-TSL (ICG: 19.3 μg/mL, DOX: 5 μg/mL) were added into different wells of 24-well plate. Using NIR laser (808 nm, 1 W/cm^2^) to irradiate the samples for 8 min, or irradiate for 5 min and immediately switch off the laser. The changes of temperature were obtained by infrared imaging camera (Ti27, Fluke, USA).

### Tumour cells

MCF-7 human breast adenocarcinoma cells were obtained from the Cell bank of Chinese Academy of Sciences (Shanghai, China) and utilized for cell studies. Cells were grown in DMEM culture medium, which contains 10% heat-inactivated FBS, 1% penicillin and 1% streptomycin, at 37 °C within humidified environment of 5% CO_2_.

### Drug release *in cells*

MCF-7 cells were seeded onto Eight-well chambered coverglasses (Lab-Tek, Nunc, USA) with a density of 1 × 10^4^/well. After overnight culturing at 37 °C, medium was replaced by fresh medium containing ICG-TSL (Containing 19.3 μg/mL ICG). After 4 h incubation and laser irradiation (808 nm, 1 W/cm^2^, 5 min), the cells were washed for thrice and stained by LysoTracker Green DND-26 (Life technologies, USA), Hoechst 33258 (Invitrogen, USA) and fixed with 4% paraformaldehyde. Finally, the subcellular localization were observed using CLSM (TCS SP5, Leica, Germany). Similarly, MCF-7 cells were seeded onto 24-well plates with a density of 4 × 10^4^/well. After overnight culturing at 37 °C, medium was replaced and cells were respectively incubated with blank medium, medium containing 4 μg/mL free DOX, and medium containing DI-TSL (containing 4 μg/mL DOX). The DOX FL of MCF-7 cells with or without laser treatment was quantitatively evaluated by Flow cytometry.

### Cellular cytotoxicity *analysis*

Cells were seeded onto 96-well plates at a density of 1 × 10^4^/well. After overnight culturing, DOX or DI-TSL were treated to MCF-7 cells and incubated for 4 h. Then immediately removed the medium, added 100 μL fresh medium, and treated without or with laser (808 nm, 1 W/cm^2^, 5 min). After 24 h further incubation, medium was removed and 20 μL MTT solutions (5 mg/mL) were added and cells were incubated for 4 h. The cytotoxicity of free DOX, DOX + laser, DI-TSL, and DI-TSL + laser was analyzed by the MTT assay.

The cytotoxicity of DOX and DI-TSL with/without laser was further evaluated by Flow Cytometry. MCF-7 cells were seeded onto 24-well plates at a density of 4 × 10^4^/well and cultured overnight. Cells were then treated with DOX or DI-TSL with/without laser (808 nm, 1 W/cm^2^, 5 min) and incubated for 4 h. Thereafter, immediately remove the medium containing drugs and cells were treated without or with laser. After 24 h incubation, MCF-7 cells were harvested, and Alexa Fluor 488 Annexin V/PI Cell Apoptosis Kit and flow cytometry (Accuri C6, BD, USA) was used for cell apoptosis analysis. Herein, Annexin V-positive and PI-negative cells were scored as apoptotic, and both Annexin V-positive and PI-positive cells were scored as late apoptotic/ necrotic cells.

### Animals and tumour model

Female BALB/c nude mice (6 weeks old, weighed 18–20 g) were purchased from Slac Laboratory Animal Co. Ltd (Hunan, China). Mice were settled in cages in groups (5 per cage), and were fed with standard water and mouse chow. All the animals received care complied with the guidelines in the Guide for the Care and Use of Laboratory Animals, and the procedures of animal experiments were approved by the Institutional Ethical Committee of Animal Experimentation of Shenzhen Institutes of Advanced Technology (Chinese Academy of Sciences). The methods were carried out strictly in accordance with governmental and international guidelines on animal experimentation. All efforts were made to minimize the usage amount of animals and the suffering during experiments according to the request of Biosafety and Animal Ethics. To set up xenograft model, 3 × 10^6^ MCF-7 cells were planted by subcutaneous injection into the flank region of the mice. Tumour volume = (tumour length) × (tumour width)^2^/2.

### *In/ex vivo* imaging and biodistribution analysis

When tumour volume of mice reached to 100–200 mm^3^, Nude mice bearing MCF-7 tumour were randomly divided into 3 groups (three per group). Mice in group 1 were intratumourally injected with 100 μL PBS as control. Mice in group 2 were intratumourally injected with 100 μL free DOX and ICG mixed aqueous solution (containing 100 μg/mL DOX and 386.3 μg/mL ICG), while mice in group 3 were injected with 100 μL DI-TSL aqueous solution (containing 100 μg/mL DOX and 386.3 μg/mL ICG). Fluorescence (FL) images and semi-quantitative results of DOX and ICG were respectively acquired at 0 h, 16 h, 24 h and 48 h after injection using *ex/in vivo* imaging system (Cri maestro, PerkinElmer, USA). The signal of DOX was obtained with a 523 nm excitation wavelength and a 560 nm filter, and ICG was collected with a 704 nm excitation wavelength and a 745 nm filter. The mice after 48 h injection were sacrificed and the major organs (heart, liver, spleen, lung, kidneys) were collected for *ex vivo* imaging and biodistribution analysis.

### Temperature increase and DOX release *in vivo*

To directly differentiate the FL distribution of free DOX/ICG and DI-TSL, we chose 16 h as investigating time point. Mice were intratumourally injected with 100 μL PBS, free DOX (containing 100 μg/mL DOX), ICG-TSL (containing 386.3 μg/mL ICG), and DI-TSL (containing 100 μg/mL DOX and 386.3 μg/mL ICG), respectively. After 16 h injection, the tumour site were irradiated by remote NIR laser (808 nm, 0.5 W/cm^2^, 5 min), and the temperature change of mice tumour were obtained by infrared imaging camera. The biodistribution of DI-TSL + laser was evaluated utilizing *in/ex vivo* imaging system. The mice bearing MCF-7 tumours were injected with 100 μL DI-TSL aqueous solution (containing 100 μg/mL DOX and 386.3 μg/mL ICG). The mice were treated with NIR laser (808 nm, 0.5 W/cm^2^, 5 min) at 16 h after intratumoural injection. Then the FL of DOX and ICG were obtained at 0 h, 16 h (before laser), 16 h (after laser), 24 h, and 48 h.

### *In vivo* apoptotic imaging

Tumour bearing mice were divided into 7 groups and were separately treated with 100 μL PBS + laser, DOX + laser, ICG-TSL, ICG-TSL + laser, DI-TSL, DI-TSL + water bath (WB, 43 °C, 5 min), and DI-TSL + laser. The concentration of DOX and ICG were normalized as 100 μg/mL and 386.3 μg/mL, and the laser (808 nm, 0.5 W/cm^2^, 5 min) or WB was treated 16 h after injection. Then the mice were intravenously injected with 100 μL Annexin-Vivo 48 h after drug injection. The images of Annexin-Vivo 750 *in vivo* of all animals were detected and acquired using *ex/in vivo* imaging system 24 h after its injection. The FL of Annexin-Vivo 750 was collected with a 740 nm excitation wavelength and a 745 nm filter. It was worth mentioned that Annexin-Vivo 750 clearance could be easily detected through kidneys and the FL in kidneys was much higher than the tumour, the kidneys were covered with a black paper to reveal the difference of Annexin-Vivo 750 in the tumour.

### *In vivo* therapeutic analysis

The antitumour effect of DI-TSL was investigated in nude mice bearing MCF-7 tumours. Tumour-bearing mice were divided into 7 groups (5 per group), and were intratumourally injected with 100 μL PBS + laser, DOX + laser, ICG-TSL, ICG-TSL + laser, DI-TSL, DI-TSL + WB (43 °C, 5 min), and DI-TSL + laser, respectively. The treatment was the same as *in vivo* apoptosis experiment. The tumour volumes and changes in body weight of mice were recorded, and mice were sacrificed by cervical dislocation under an anesthetic status after the experiments (15 d post-treatment). Mice with tumour volume exceeding 1000 mm^3^ were euthanatized according to the animal protocols. To further detect the safety of drugs, the major organs of mice 15 d after treatment were collected and stained with H&E.

### Statistical analysis

All the results were showed as mean or mean ± standard deviation. Students’s *t*-test was utilized to evaluate the significance in two groups, and one-way ANOVA was used among multiple groups. Herein, single asterisk (*) indicated *P* < 0.05, which was considered as significant difference, and double asterisk (**) indicated *P* < 0.01, which was considered as very significant difference.

## Additional Information

**How to cite this article**: Zhao, P. *et al.* NIR-driven Smart Theranostic Nanomedicine for On-demand Drug Release and Synergistic Antitumor Therapy. *Sci. Rep.* **5**, 14258; doi: 10.1038/srep14258 (2015).

## Supplementary Material

Supplementary Information
