# Supplementary material for: NIR-driven Smart Theranostic Nanomedicine for On-demand Drug Release and Synergistic Antitumour Therapy
Source: Sci Rep. 2015 Sep 24;5:14258. doi: 10.1038/srep14258 (PMC4585834; doi:10.1038/srep14258)
Supplement: Supplementary Information [file srep14258-s1.pdf]

Supplementary Information

NIR-driven Smart Theranostic Nanomedicine for On-demand Drug Release and Synergistic Antitumour Therapy

Pengfei Zhao, Mingbin Zheng[‡]*, Zhenyu Luo, Ping Gong, Guanhui Gao, Zonghai Sheng, Cuifang Zheng, Yifan Ma, and Lintao Cai*[‡]

Supplementary Table 1. Characterization of DI-TSL and ICG-TSL. The data were shown as mean ± SD (n = 3).

Type	Size (nm)	Surface potential (mV)	DOX-EE (%)	ICG-EE (%)	DOX-LE (%)	ICG-LE (%)
DI-TSL	43.8±3.4	-23.5±2.9	27.1±1.6	28.1±2.6	3.5±0.1	3.9±0.4
ICG-TSL	42.2±5.3	-26.5±0.2	——	40.9±3.3	——	6.4±0.2

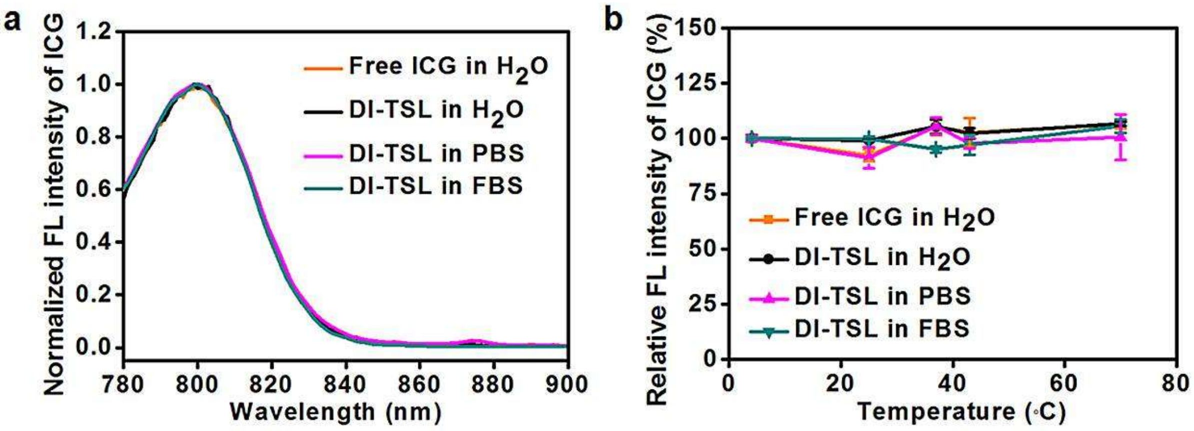

Supplementary Figure 1. Stability of ICG FL in different solutions and temperatures. a) ICG fluorescence (FL) spectra of free ICG in ultrapure water and DI-TSL in ultrapure water, PBS, and FBS. b) Relative ICG FL intensity of free ICG in ultrapure water and DI-TSL in ultrapure water, PBS, and FBS at different temperatures.

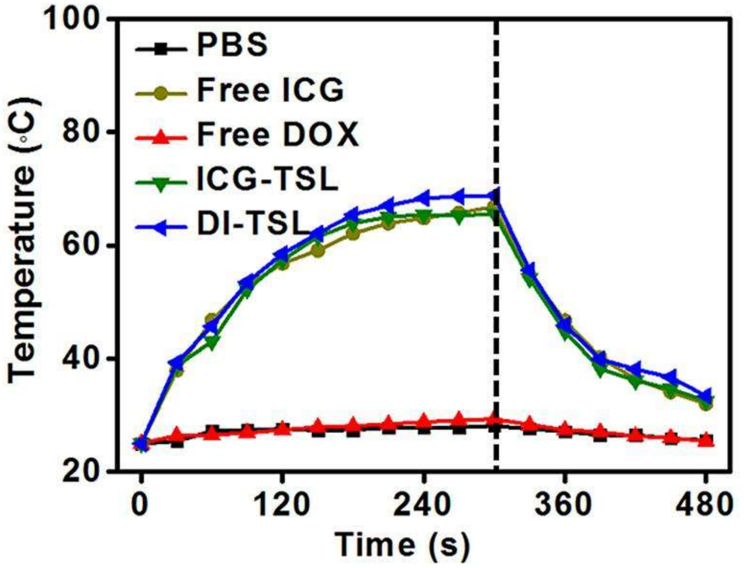

Supplementary Figure 2. Temperature rising profiles of PBS, free ICG, free DOX, ICG-TSL, and DI-TSL under continuous laser irradiation (808 nm, 1 W/cm^2). And the laser was switched off at 5 min.

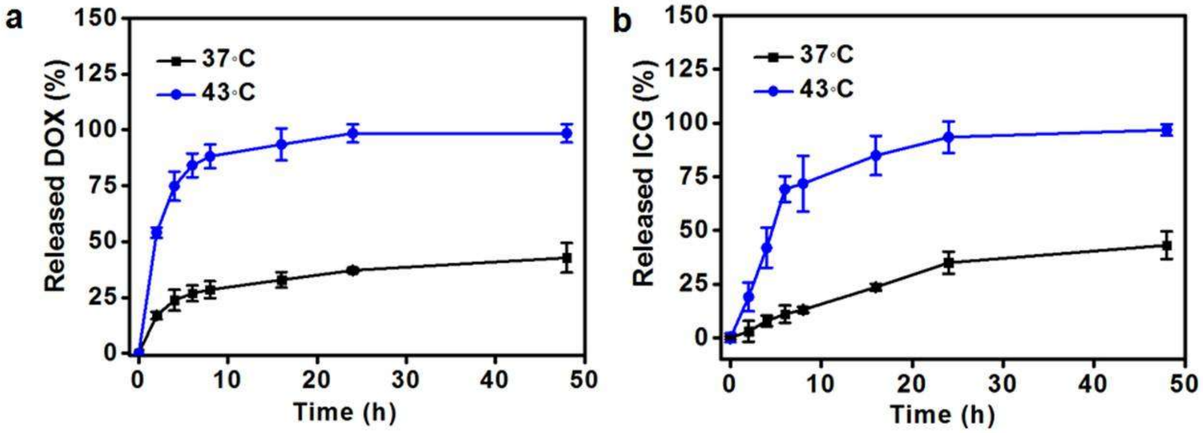

Supplementary Figure 3. Drug release profiles of DI-TSL at 37℃ or 43℃ within 48 h. a) DOX release from DI-TSL at 37℃ or 43℃ within 48 h. b) ICG release from DI-TSL at 37℃ or 43℃ within 48 h.

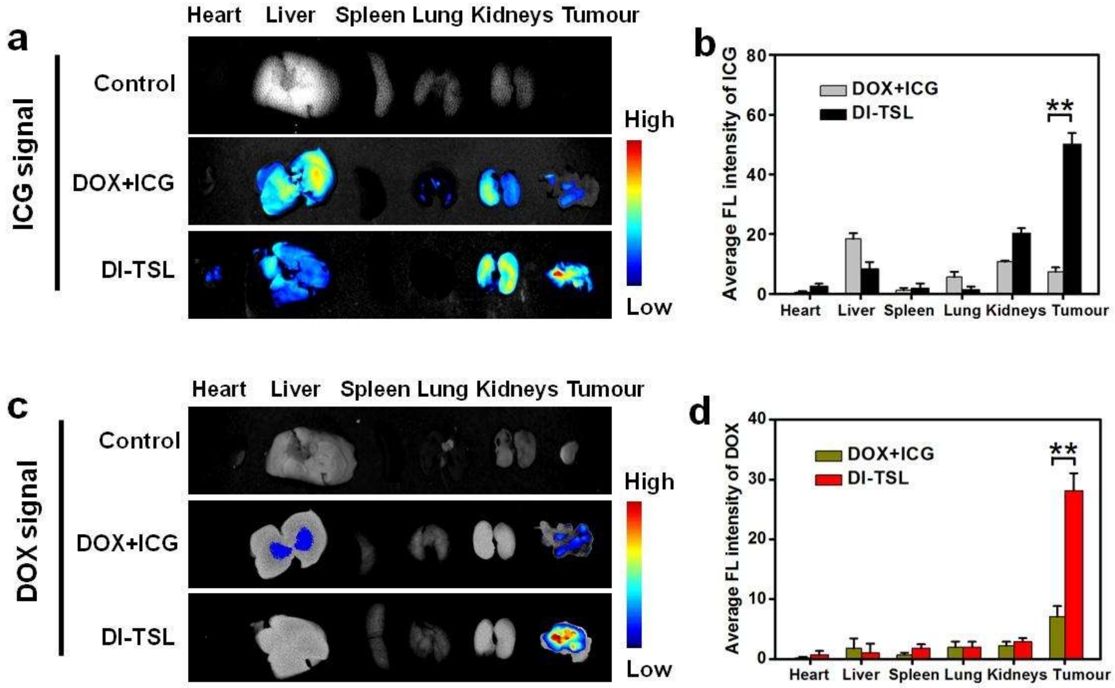

Supplementary Figure 4. *Ex vivo* imaging of DOX/ICG FL in major organs and tumours of nude mice bearing MCF-7 tumours 48 h after intratumoural injection of PBS, DOX + ICG, and DI-TSL. a) ICG FL images of the major organs and tumours of mice 48 h after intratumoural injection of PBS, DOX + ICG, and DI-TSL. b) Averaged ICG FL intensities of corresponding individual organs and tumours of mice in PBS, DOX + ICG, and DI-TSL groups 48 h after injection (n = 3). $^{**}P < 0.01$. c) DOX FL images of the major organs and tumours of mice 48 h after intratumoural injection of PBS, DOX + ICG, and DI-TSL. d) Quantitative DOX FL intensities of free DOX and DI-TSL in nude mice 48 h after injection determined by averaged DOX FL intensity of individual organs and tumours (n = 3). $^{**}P < 0.01$.

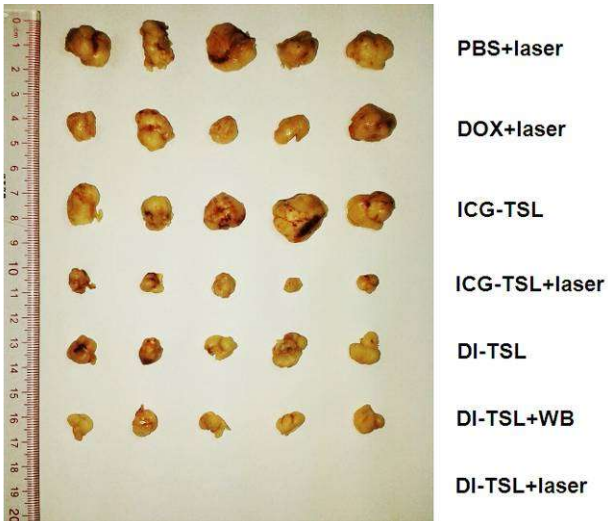

Supplementary Figure 5. Photos of the tumours extracted from the mice bearing MCF-7 tumour 15 d after treatment as indicated. DI-TSL exhibited completely tumour eradication, and tumours treated with DOX + laser, ICG-TSL + laser, DI-TSL, or DI-TSL + WB (water bath) displayed a tumour re-growth.

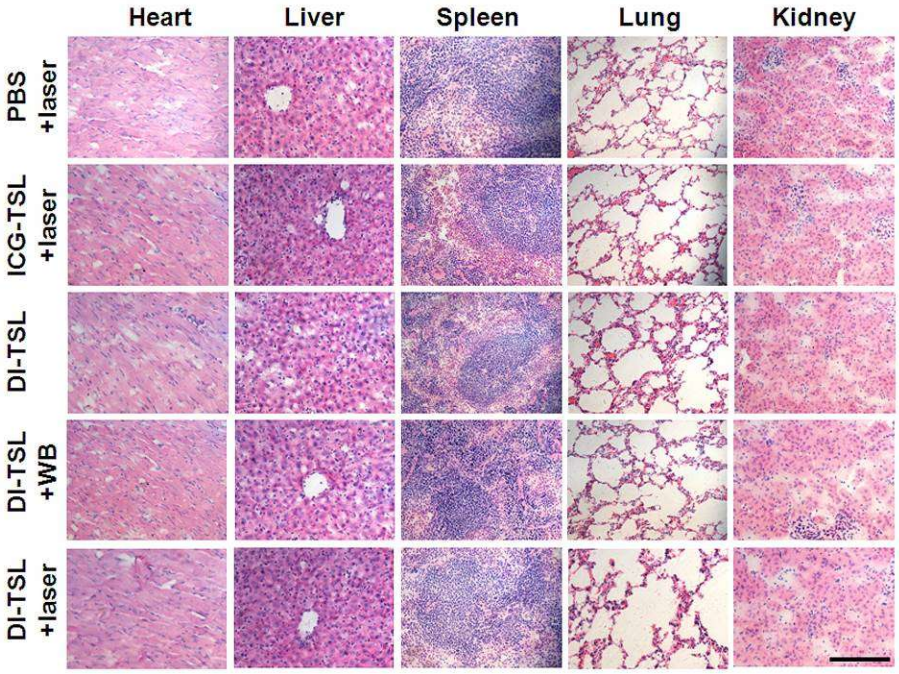

Supplementary Figure 6. H&E stained images of major organs collected from different groups of mice 15 d after treatment (scale bar = 100 μm). Overall treatments displayed excellent safety to heart, liver, spleen, lung and kidney owing to the low dosage of DOX used in the treatments, which could be well tolerated by nude mice.
